# A protocol for a randomized clinical trial assessing the efficacy of hypertonic dextrose injection (prolotherapy) in chronic ankle instability

**DOI:** 10.1186/s13063-022-07037-7

**Published:** 2022-12-29

**Authors:** Regina Wing Shan Sit, Ricky Wing Keung Wu, Samuel Ka Kin Ling, Patrick Shu Hang Yung, Bo Wang, Dicken Cheong Chun Chan, Benjamin Hon Kei Yip, Samuel Yeung Shan Wong, Kenneth Dean Reeves, David Rabago

**Affiliations:** 1 The Jockey Club School of Public Health and Primary Care, Chinese University of Hong Kong, Hong Kong, China; 2 The Hong Kong Insititute of Musculoskeletal Medicine, Hong Kong, China; 3 The Department of Orthopedic and Traumatology, Chinese University of Hong Kong, Hong Kong, China; 4 Roeland Park, USA; 5 Department of Family and Community Medicine, Pennsylvania State University, Hershey, USA

**Keywords:** Hypertonic dextrose injection, Prolotherapy, Chronic ankle instability

## Abstract

**Background:**

Lateral ankle sprain (LAS) is a common injury. Conservative care is not uniformly effective. Chronic ankle instability (CAI) results in up to 70% of patients with LAS in the physically active population. LAS, together with subsequent osteochondral lesions and pain in many patients, leads to the development of post-traumatic osteoarthritis, resulting in a substantial direct and indirect personal and societal health burden. Dextrose prolotherapy (DPT) is an injection-based therapy for many chronic musculoskeletal conditions but has not been tested for CAI. This protocol describes a randomized controlled trial to test the efficacy of DPT versus normal saline (NS) injections for chronic ankle instability (CAI).

**Methods and analysis:**

A single-center, parallel-group, randomized controlled trial will be conducted at a university-based primary care clinic in Hong Kong. A total of 114 patients with CAI will be randomly allocated (1:1) to DPT and NS groups. The primary outcome will be the Cumberland Ankle Instability Tool scores at 1 year. The secondary outcomes will be the number of re-sprains in 1 year, the Star Excursion Balance Test, the 5-level of EuroQol 5-dimension questionnaire, and the Foot and Ankle Ability Measure. All outcomes will be evaluated at baseline and at 16, 26, and 52 weeks using a linear mixed model.

**Discussion:**

We hypothesized the DPT is a safe, easily accessible, and effective treatment for patients with CAI. This RCT study will inform whether DPT could be a primary non-surgical treatment for CAI.

**Trial registration:**

Chinese Clinical Trial Registry ChiCTR2000040213. Registered on 25 November 2020.

## Introduction

Chronic ankle instability (CAI) is a common condition and it often results from lateral ankle sprain (LAS), especially in the physically active population [1]. Up to 70% of individuals have some type of residual symptoms, such as recurrent ankle sprains and perceived instability [2, 3]. A recent large longitudinal prospective study examined the onset of sensorimotor deficits after LAS which reported that CAI often begins shortly after the acute sprain injury, with a prevalence of 40% in 1 year after a first-time LAS, and is associated with long-term postural control deficits and multiple aberrant movement patterns [4]. Chronic joint instability together with subsequent osteochondral lesions eventually leads to the development of post-traumatic osteoarthritis [5], along with reduced physical activity levels and health-related quality of life [6, 7]. While the direct costs for treatment of isolated LAS are relatively low, compounding these are indirect costs from follow-up care, loss of productivity and leisure time, and care of long-term consequences of CAI [8, 9].

The lateral ligament complex of the ankle is composed of 3 ligaments: anterior talofibular ligament (ATFL), posterior talofibular ligament, and calcaneofibular ligament. The ATFL originates from the anterior aspect of the distal fibula, approximately 1 cm from the tip of the lateral malleolus, and it inserts on the talar body just anterior to the articular surface. The ATFL is the weakest ligament affected in most LAS injuries, and its residual laxity is the major underlying pathology of CAI [10]. Initial treatment of CAI is always conservative, which includes the use of an ankle brace or ankle tape but has no effect on proprioceptive acuity [11]. Exercise-based rehabilitation improves ankle stability in CAI, yet no consensus has been reached on the optimal exercise content and training volume [12]. Surgical interventions are often reserved for complete rupture of the lateral ligament complex or CAI which failed conservative treatment, but have associated surgical risks and high cost [13]. Therefore, the search for other minimally invasive therapies for CAI is necessary, among which injection therapy may be an option.

Dextrose prolotherapy (DPT) is an injection used to treat chronic painful musculoskeletal conditions through stimulation of tissue proliferations [14–17]. It consists of injections of hypertonic dextrose with a concentration between 10 and 25% to ligament-bone or tendon-bone system or in the intra-articular space, which is performed repeatedly at the established interval. Pre-clinical studies have suggested its mechanism of actions through initiation of a temporary inflammatory reaction with cytokine-induced tissue repair, granulation, soft tissue proliferation, and extracellular matrix remodeling [18–20]. The exposure of human tenocytes to DPT had been shown to induce an inflammatory response through the up-regulation of pro-inflammatory mediators [21]. In mouse models, DPT was found to stimulate fibroblast proliferation through activation of the Erk Pathway [22]. Soft tissues such as ligaments and collagen bundles also demonstrate a larger cross-sectional area and a better tensile strength after DPT [23–25].

Clinically, DPT has been used to treat conditions with specific soft tissue-mediated joint instability. Its strengthening effect on anterior cruciate ligament laxity had been proposed in a small clinical trial with patients having knee osteoarthritis [26, 27]. In a prospective single-arm study, patients with sacroiliac joint instability receiving DPT injections to the dorsal interosseous ligament have shown functional improvement for up to 24 months [28]. DPT provided significant relief of pain in patients with sacroiliac joint instability, and with longer effect than steroid injections [29]. Overall, rigorous evidence supporting DPT for ligament injuries is lacking; trials are limited in number, often of short duration, and lack methodological rigor.

This study aims to assess the clinical efficacy of DPT compared to normal saline injections among individuals with CAI in a randomized controlled trial (RCT). Our hypotheses are that DPT, compared with blinded saline injection, will result in improved self-reported ankle instability, function, and quality of life; reduced total number of re-sprains; and improved objectively assessed function over 1 year.

## Methods and analysis

**Fig. 1:**
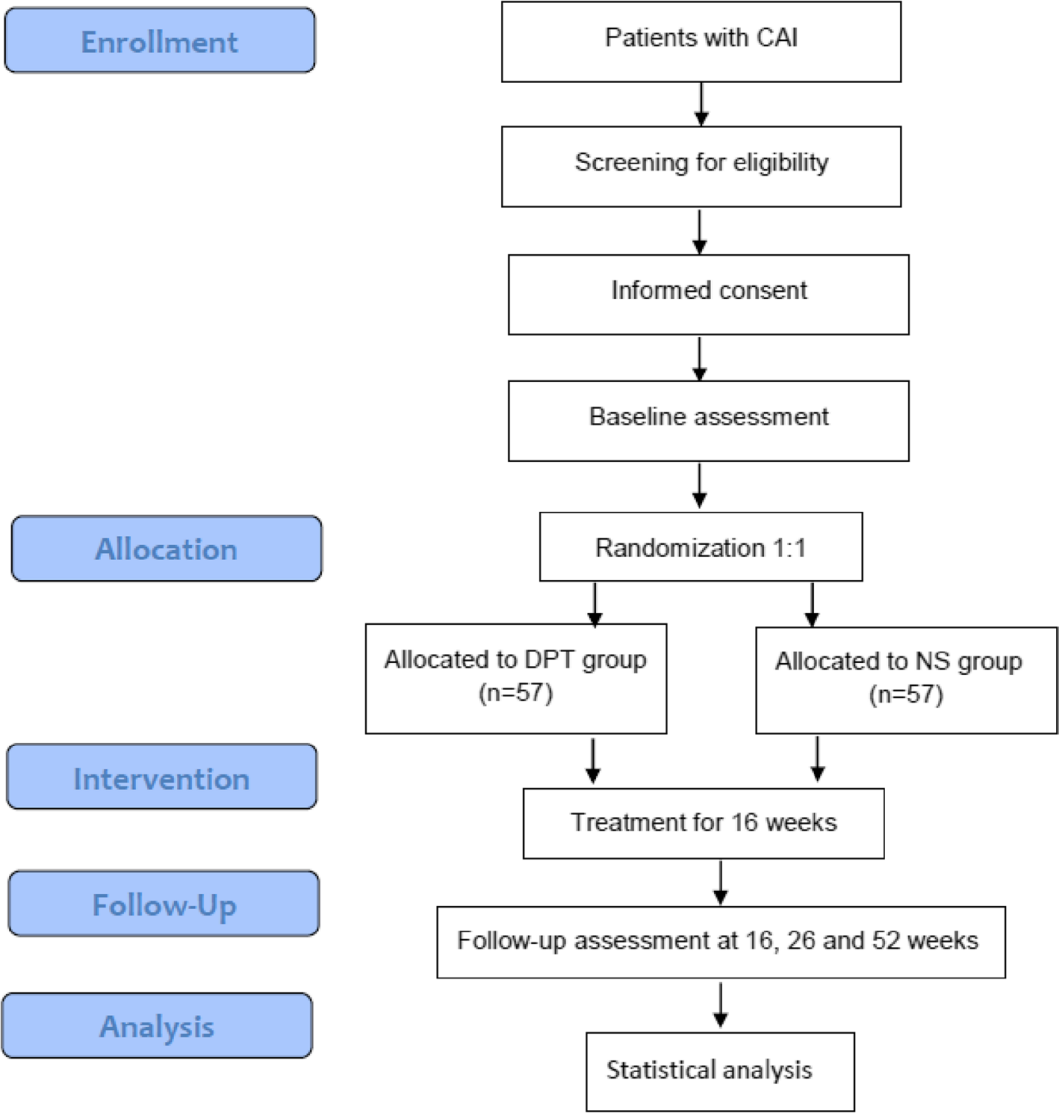
Flow chart of the study

**Fig. 2:**
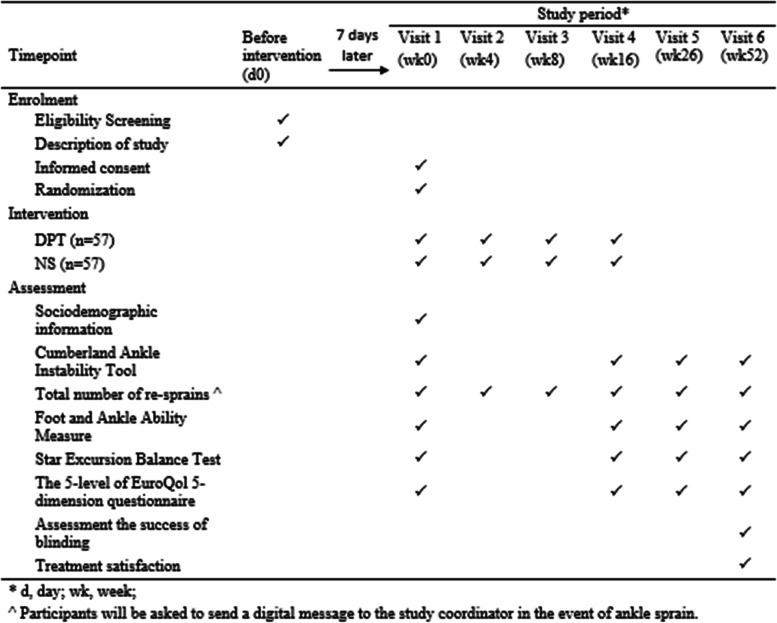
SPIRIT figure: schedule of enrollment, intervention, and assessment

The study is a 52-week, two-arm, parallel, superiority, RCT. The study flow has been summarized in Fig. 1. The Standard Protocol Items: Recommendations for Interventional Trials (SPIRIT) schedule of enrolment, interventions, and assessments is illustrated in Fig. 2. We follow the International Ankle Consortium Position Statement regarding the eligibility criteria for CAI and ultrasound evidence of ATFL laxity [30]. The inclusion criteria include age ≥ 18 years old; a diagnosis of CAI, defined as having at least 1 significant ankle sprain at least 12 months prior to study enrolment, defined as sprain associated with inflammatory symptoms (pain, swelling, etc.) and created at least 1 interrupted day of desired physical activity; at least 2 episodes of “giving way” in the 6 months prior to study enrollment; and a Cumberland Ankle Instability Tool (CAIT) score ≤ 24. Eligibility will be further clinically determined by the principal investigator (PI). Eligible participants will have a positive anterior drawer test (anterior talofibular ligament involvement) [31] and a length change of the ATFL under stress (maximum anterior draw position) and at rest of >20% [32]. Ultrasound has a high sensitivity of 97.7% and a high specificity of 92.3% for the detection of chronic ATFL injury [33] and is a recommended imaging modality for CAI.

The exclusion criteria include acute ankle sprain ≤ 6 months; complete tear of ATFL confirmed by ultrasound or magnetic resonance imaging (MRI); a history of previous surgeries to musculoskeletal structures (i.e., bones, joint structures, nerves) in either lower extremity; acute injury to musculoskeletal structures of other joints of the lower extremity in the previous 3 months that impacted joint integrity and function (i.e., sprains, fractures), resulting in at least 1 interrupted day of desired physical activity; a history of a fracture in either lower extremity requiring realignment [30]; high ankle sprain, generalized ligament laxity [34]; pregnancy; anticoagulant therapy; prior ATFL or ankle injections within 3 months; inflammatory or post-infectious ankle arthritis, such as clinically diagnosed rheumatoid arthritis, gouty arthritis, psoriatic arthritis, and septic arthritis; history of corn allergy [35, 36]; and co-morbidity or lifestyle preventing participation in the study protocol.

### Recruitment and consent

Participants will be recruited through social media and at primary care clinics in the New Territories East Region of Hong Kong. The study site is a university primary care clinic. After confirming the eligibility of the participants, the principal investigator will take approximately 15 min to describe the study goals, procedures, activities, and possible alternatives and answer all questions. Following this, interested candidates will be given 7 days to consider enrolment. The research assistant will then call the candidates for a second visit when written informed consent will be signed. After enrolment, participants will receive a study identification number and undergo baseline data collection.

### Randomization and allocation concealment

Randomization will be done in a 1:1 ratio using the Random Allocation Software [37]. The allocation sequence will be concealed from the researcher enrolling and assessing participants through the use of sequentially numbered, opaque, sealed envelopes [38]. The treatment allocation process starts when the investigator calls the personnel keeping the envelopes. The computer database is designed in such a way that treatment allocation cannot be changed after randomization. Each participant will receive the envelope and they will be asked to sign it. These envelopes will be kept by a person not involved in the care or evaluation of patients, or in the data collection or analysis. The envelopes will only be opened at 52 weeks after study completion.

### Blinding of participants and personnel

Two registered nurses not involved in participants’ care will prepare the syringes with dextrose or NS identified only by study identification numbers. The syringes will be wrapped in aluminum foil to mask the solutions. The principal investigator and the study coordinator will therefore be blinded to the group status of all subjects. The physician who conducts the injections will be blinded to the allocation group; he is also prohibited from communicating with participants. Dextrose and saline solutions are odorless and identical in color and viscosity. Participants will be blinded to their group status, knowing only their randomization group number.

### Blinding in outcome assessment and data analysis process

All data collection will be performed by trained research assistants blinded to the allocation status of the patients via face-to-face interviews. They will receive rigorous training in standardized data collection procedures. Data entry personnel external to the research team will be employed to perform data entry such that the statistician can analyze data without the need to refer to allocation information.

### Intervention descriptions

**Fig. 3:**
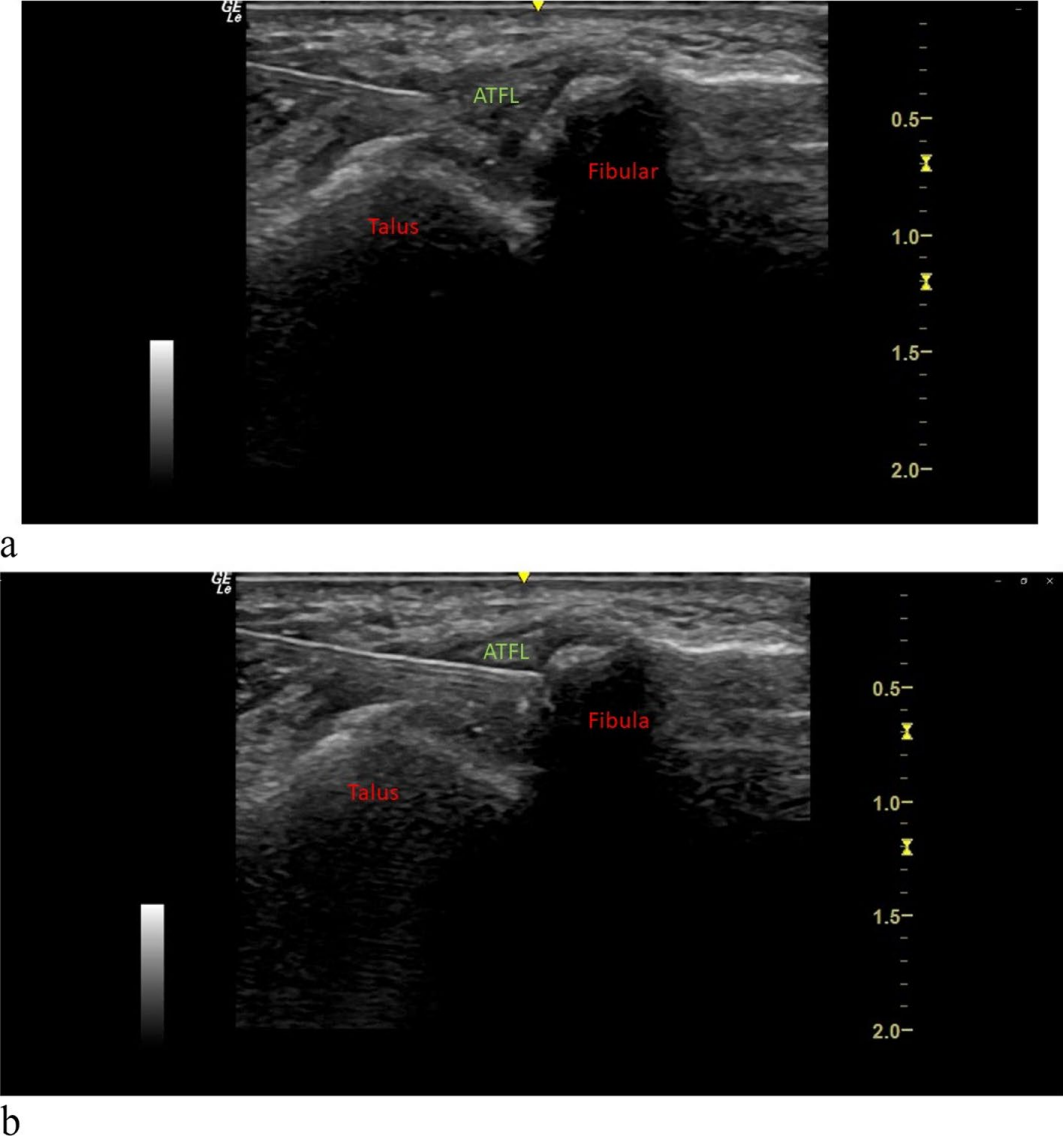
**a** Injection of anterior talofibular ligament, in-plane approach under ultrasound guidance (talus end). **b** Injection of anterior talofibular ligament, in-plane approach under ultrasound guidance (fibula end)

The injection procedures will be conducted under an aseptic technique by trained physicians. Following sterile preparation and injection of 1 ml 2% xylocaine as local anesthetic blebs, participants will be injected under ultrasound guidance with 25-gauge 1.5-inch needle directed to the ATFL attachment on the talus and fibula using a linear probe with an in-plane approach and peppering technique (Fig. 3a, b). We will follow the conventional prolotherapy technique prolotherapy adapted from standard prolotherapy texts to inject the ATFL at weeks 0, 4, 8, and 16 [39].

In the intervention group, syringes will be loaded with 5 ml of 15% dextrose (D15), prepared by mixing 1.5ml 50% dextrose and 3.5 ml sterile water. In the control group, syringes will be loaded with 5 ml normal saline. In case of pain flares after injection, the subsequent injection will be commenced after the flare is subsided, or at 1 month. If participants display allergic symptoms to the injected solution, therapy will be terminated but participants will continue to be followed in their allocated group until the end of the study.

Participants will be observed for 10 min after each injection. They will be advised to take acetaminophen (500 to 1000 mg every 4 to 6 h as needed) and avoid non-steroidal anti-inflammatory drugs in the first week after injection, which may interfere with the DPT mechanism of action. Participants will be instructed on post-injection care and slow ramp-up of activity.

Participants in both groups will receive an online video (https://youtu.be/99VxZoUtEVQ) which demonstrates proprioceptive training of the foot and ankle. The video includes the practice of 6 land-based exercises (i.e., one-legged knee flexion, toe stand, one-legged stance, runner pose, cross-leg sway, and toe walk). The exercise frequency is 3 times per week, consistent throughout the study period [40, 41].

Co-interventions will be discouraged but allowed in both groups, such as conventional medication, physical therapy, acupuncture, herbal medicine, over-the-counter drugs, and other active treatments. The use of co-interventions will be tracked during the study period and assessed as co-variates.

### Baseline and outcome measurement at 0, 16, 26, and 52 weeks

Primary outcome:

1. The primary outcome will be Cumberland Ankle Instability Tool (CAIT) assessed at 52 weeks. The CAIT is a 9-item 30-point scale, with a lower score indicating more functional ankle instability [42]. CAIT is a widely recommended discriminative instrument for the identification of CAI with good responsiveness [43]. A validated Chinese version will be used in this study [44].

Secondary outcomes:

2) The total number of re-sprains in 1-year follow-up as assessed by a study-specific injury registry. Participants will be asked to send a digital message to the study coordinator in the event of an ankle sprain. An ankle sprain is defined as an ankle injury occurring as a result of sports participation or activities of daily living and which causes one or more of the following: participants have to stop the sports activity, and/or cannot (fully) participate in the next planned sports activity, and/or cannot go to work/school the next day, and/or needs medical attention (ranging from onsite care by, e.g., general practitioner, to personal care by, e.g., sports physician) [45].
3) Self-reported function as assessed by the 29-item Foot and Ankle Ability Measure (FAAM): it includes two subscales: activity of daily living (21 items) and sports (8 items). The total score ranges from 0 to 100; a higher value means greater physical function [46]. Its validity has been demonstrated in CAI [47], and a validated Chinese version will be used in this study [48].
4) Objectively measured balance, as assessed by the Star Excursion Balance Test (SEBT) [49]. To perform the SEBT, patients will stand on a single leg with the study limb placed at the center of a grid with 8 lines extending at 45° increments. They then attempt to reach the furthest possible point with one leg while maintaining balance on the contralateral leg. During the performance of SEBT, only the anterior (A), posterior-medial (PM), and posterior-lateral (PL) directions will be used. The distance from the center of the grid to the maximal reach point will be measured in centimeters with a measuring tape. Participants will be allowed 4 tries in each direction to get familiarized with the test and will be allowed to perform 3 reaches in each direction with 30-s rest between trials. The starting position will be randomly assigned to avoid a learning effect. The reaches in each direction will be normalized with the leg length. Lower limb length will be determined, with the subject lying supine, by measuring from the anterior superior iliac spine to the distal end of the medial malleolus. The SEBT composite score will be calculated by dividing the sum of the 3 reaches distances in the 3 directions by 3 times the limb length (LL) of the individual, then multiplied by 100 {[(A+PM+PL)/(LL×3)] }×100.
5) Health-related quality of life as assessed by the 5-level of EuroQol 5-dimension questionnaire (EQ-5D-5L) [50]. Patients will be asked to self-rate their current health state on a visual analogue scale (EQ5D VAS) from 0 to 100. The EQ-5D-5L assesses five dimensions of current health: mobility, self-care, usual activities, pain/discomfort, and anxiety/depression (EQ5D index). A local study has evaluated the value set of the Hong Kong Chinese EQ-5D-5L questionnaire [51].
6) To assess the success of blinding, participants will be asked to guess their group status at 52 weeks.
7) Treatment satisfaction will be assessed at 52 weeks by a question “Are you satisfied with the treatment received?”.

Sociodemographic data such as age, sex, occupation, and imaging confirmed osteochondral lesions or post-traumatic osteoarthritis will be collected and controlled as con-founders.

### Sample size calculation

Since no previous studies have evaluated the role of DPT in CAI, we estimated our sample size based on the paper by Weight et al. which calculated the minimal clinical important difference (MCID) of CAIT in a group of athletes with CAI. In this paper, the mean change of CAIT after focused ankle rehabilitation interventions, i.e., wobble board balance training and resistance tubing strengthen training, was 4.4 (SD 5.1) whereas the control group without any intervention was −1.4 (SD 3.4), yielding Cohen *D* of 1.34 [52]. Since the sample size of Weight’s et al. was small (*n*=50), the assessment period was short (only 4 weeks), and their participants were very young (mean age 21.5), we assumed a more conservative effect size for DPT of 0.60. With alpha 0.5 and power 85%, the calculated sample size will be 102. With the dropout rate of 10%, the total sample size will be 114, with 57 at each arm.

### Statistical analysis

We will conduct an analysis of covariance (ANCOVA) to compare the CAIT score at 52 weeks between the intervention groups with adjustment of the baseline CAIT score. For the secondary analysis, we will use ANCOVA for other secondary continuous outcome measures and logistic regression for the binary outcome. To study the trend, we will conduct generalized estimating equations models (GEE) for both primary and secondary outcomes following the intention to treat principle, i.e., all available data will be analyzed according to the group they are randomly assigned. The use of GEE also provides the means to include subjects with incomplete and use all available data to assess the treatment effect over time. Given the longitudinal nature of the clinical trial data, we assume the autoregressive covariance structure will be the best fit for the data, but the statistical fitness by using other covariance structures will also be evaluated [53].

With a clearly defined target population, efficacy and safety outcomes, and convenient data collection procedures, our trial should realize the goal of maximizing the number of participants who are maintained on the protocol-specified intervention until the outcome data are collected. We will use multivariate imputation using chained equations (MICE) to incorporate auxiliary information about the missing data. The imputation model will include prerequisite variables in the data analysis. About 10 iterations will be conducted in each imputation process with more iterations to be considered until the chain reaches convergence [54]. Twenty completed datasets will be imputed with the use of the chain equations. Rubin’s rule will be applied to combine the effect estimates [55]. This approach provides estimated standard errors and *P* values that incorporate missing-data uncertainty. We will use the statistical package SPSS version 24 (SPSS Inc., IL, USS). All statistical tests will be two-tailed with a significant level of 0.05.

## Safety monitoring

Participants will have a diary to document any discomfort after each injection. Potential injection risks include bleeding, infection, and nerve injuries. Participants are advised to call or WhatsApp the study coordinator if they are uncertain whether the discomfort is related to the injections. Standardization forms will be used for monitoring and reporting side effects and adverse events. The principal investigator will be present in case of a significant adverse event. The principal investigator will report serious adverse events to the ethics committee within 24 h, and annual reports summarizing adverse events will be submitted to the Drug Office of the Department of Health, Hong Kong Administrative Region. A “stopping rule” will be applied to participants who underwent ankle surgery or experienced severe adverse events during the study period [56].

### Data monitoring committee

A data monitoring committee is formed for data quality assurance, monitoring, and reporting. The team is composed of a senior clinical researcher with rich experience in clinical trials, an Orthopedic surgeon, and a Family Medicine Specialist. A research assistant and a nurse will also be members of the QA team. Interim data monitoring will be done by a biostatistician who is not a co-investigator to monitor the quality of the study every 3 months.

### Monitoring and quality assurance

The coordinating center is composed of the principal investigator (physician), nurse, and research team member and are responsible for monitoring the whole trial process. They will meet every week to discuss the trial process and any problem that was encountered during the trial. The data will be stored in a password-protected cloud server and only the principal investigator and research team member have access to the dataset.

## Dissemination

The findings of this RCT will be disseminated through presentations at public lectures, scientific institutions, and meetings and through peer-reviewed scientific articles. The de-identified data with statistical codes can be obtained from the corresponding author upon reasonable request.

## Discussion

Apart from foot and ankle rehabilitation on muscle strengthening, balance, proprioception, and correction of biomechanics, conservative treatment options for CAI are limited, and surgery is an effective option when conservative treatment fails; drawbacks include the risk of surgical complications and relatively higher costs [57]. DPT, with reported pain and function effects for other musculoskeletal soft tissue injuries, and potential regenerative effects on soft tissue injury, may be an alternative treatment option to those who failed conservative management of CAI. Dextrose is inexpensive and readily accessible, and the injection of ATFL can be easily performed by trained physicians with or without ultrasound. The efficacy of DPT, as measured by validated self-reported instability, function and quality-of-life, and objectively assessed-functional outcomes in this trial, will further inform its role in clinical practice.

## Trial status

The first approved protocol was on 23 October 2019 and the latest approved protocol is version 4 on 11 March 2022, by the Joint Chinese University of Hong Kong – New Territories East Cluster Clinical Research Ethics Committee. Recruitment began in February 2021 and was completed in December 2021. The last patient visit will be expected on 5 March 2023. The protocol was initially submitted before the completion of recruitment; however, the review process was much delayed due to COVID-19 and other administrative issues. The protocol is now submitted to *Trials*, and the last patient visit for outcome assessment will be on 5 March 2023.

## Data Availability

The de-identified data with statistical codes and the relevant results in this study will be shared through academic conferences and scientific papers. The datasets can be obtained from the corresponding author upon reasonable request.

## Abbreviations

LAS: Lateral ankle sprain
CAI: Chronic ankle instability
DPT: Dextrose prolotherapy
NS: Normal saline
ATFL: Anterior talofibular ligament
RCT: Randomized controlled trial
CAIT: Cumberland Ankle Instability Tool
PI: Principal investigator
D15: 15% dextrose
FAAM: Foot and Ankle Ability Measure
SEBT: Star Excursion Balance Test
A: Anterior
PM: Posterior-medial
PL: Posterior-lateral
LL: Limb length
EQ-5D-5L: 5-Level of EuroQol 5-dimension questionnaire
VAS: Visual analogue scale
MCID: Minimal clinical important difference
ANCOVA: Analysis of covariance
GEE: Generalized estimating equations models
SD: Standard deviation

## References

[1] Herzog MM, Kerr ZY, Marshall SW, Wikstrom EA (2019). Epidemiology of ankle sprains and chronic ankle instability. J Athl Train.

[2] Anandacoomarasamy A, Barnsley L (2005). Long term outcomes of inversion ankle injuries. Br J Sports Med.

[3] Konradsen L, Bech L, Ehrenbjerg M, Nickelsen T (2002). Seven years follow-up after ankle inversion trauma. Scand J Med Sci Sports.

[4] Doherty C, Bleakley C, Hertel J, Caulfield B, Ryan J, Delahunt E (2016). Recovery from a first-time lateral ankle sprain and the predictors of chronic ankle instability: a prospective cohort analysis. Am J Sports Med.

[5] Golditz T, Steib S, Pfeifer K (2014). Functional ankle instability as a risk factor for osteoarthritis: using T2-mapping to analyze early cartilage degeneration in the ankle joint of young athletes. Osteoarthr Cartil.

[6] Hubbard-Turner T, Wikstrom EA, Guderian S, Turner MJ (2015). An acute lateral ankle sprain significantly decreases physical activity across the lifespan. J Sports Sci Med.

[7] Arnold BL, Wright CJ, Ross SE. Functional ankle instability and health-related quality of life. J Athl Train. 2011;46(6):634–41. 10.4085/1062-6050-46.6.634 PMC3418941 22488189

[8] Gerber JP, Williams GN, Scoville CR, Arciero RA, Taylor DC (1998). Persistent disability associated with ankle sprains: a prospective examination of an athletic population. Foot Ankle Int.

[9] Waterman BR, Owens BD, Davey S, Zacchilli MA, Belmont PJ (2010). The epidemiology of ankle sprains in the United States. J Bone Joint Surg Am.

[10] Guillodo Y, Varache S, Saraux A (2010). Value of ultrasonography for detecting ligament damage in athletes with chronic ankle instability compared to computed arthrotomography. Foot Ankle Spec.

[11] Raymond J, Nicholson LL, Hiller CE, Refshauge KM (2012). The effect of ankle taping or bracing on proprioception in functional ankle instability: a systematic review and meta-analysis. J Sci Med Sport.

[12] Bleakley CM, Taylor JB, Dischiavi SL, Doherty C, Delahunt E (2019). Rehabilitation exercises reduce reinjury post ankle sprain, but the content and parameters of an optimal exercise program have yet to be established: a systematic review and meta-analysis. Arch Phys Med Rehabil.

[13] de Vries JS, Krips R, Sierevelt IN, Blankevoort L, Van Dijk C. Interventions for treating chronic ankle instability. Cochrane Database Syst Rev. 2011;8:1–13. 10.1002/14651858.CD004124.pub3. 10.1002/14651858.CD004124.pub3 PMC13254623 21833947

[14] Sit RW, Chung V, Reeves KD (2016). Hypertonic dextrose injections (prolotherapy) in the treatment of symptomatic knee osteoarthritis: a systematic review and meta-analysis. Sci Rep.

[15] Zhu M, Rabago D, Chung VC-h, Reeves KD, Wong SY-S, Sit RW-S (2022). Effects of hypertonic dextrose injection (prolotherapy) in lateral elbow tendinosis: a systematic review and meta-analysis. Arch Phys Med Rehabil.

[16] Sit RW-S, Reeves KD, Zhong CC (2021). Efficacy of hypertonic dextrose injection (prolotherapy) in temporomandibular joint dysfunction: a systematic review and meta-analysis. Sci Rep.

[17] Chutumstid T, Susantitapong P, Koonalinthip N. Effectiveness of dextrose prolotherapy for the treatment of chronic plantar fasciitis: a systematic review and meta-analysis of randomized controlled trials. PM R. 2022. https://doi.org/1002/pmrj.12807. 10.1002/pmrj.12807 35338597

[18] Reeves KD, Sit RW, Rabago DP (2016). Dextrose prolotherapy: a narrative review of basic science, clinical research, and best treatment recommendations. Phys Med Rehabil Clin N Am.

[19] Sanderson LM, Bryant A (2015). Effectiveness and safety of prolotherapy injections for management of lower limb tendinopathy and fasciopathy: a systematic review. J Foot Ankle Res.

[20] Han DS, Lee CH, Shieh YD, et al. A role for substance P and acid-sensing ion channel 1a in prolotherapy with dextrose-mediated analgesia in a mouse model of chronic muscle pain. Pain. 2022;163(5):e622–33. 10.1097/j.pain.0000000000002440 34382604

[21] Ekwueme EC, Mohiuddin M, Yarborough JA (2017). Prolotherapy induces an inflammatory response in human tenocytes in vitro. Clin Orthop Relat Res.

[22] Woo MS, Park J, Ok S-H (2021). The proper concentrations of dextrose and lidocaine in regenerative injection therapy: in vitro study. Korean J Pain.

[23] Jensen KT, Rabago D, Best TM, Patterson JJ, Vanderby R (2008). Longer term response of knee ligaments to prolotherapy in a rat injury model. Am J Sports Med.

[24] Yoshii Y, Zhao C, Schmelzer JD, Low PA, An K-N, Amadio PC (2009). The effects of hypertonic dextrose injection on connective tissue and nerve conduction through the rabbit carpal tunnel. Arch Phys Med Rehabil.

[25] Yoshii Y, Zhao C, Schmelzer JD, Low PA, An K-N, Amadio PC (2014). Effects of multiple injections of hypertonic dextrose in the rabbit carpal tunnel: a potential model of carpal tunnel syndrome development. Hand..

[26] Reeves KD, Hassanein K (2000). Randomized prospective double-blind placebo-controlled study of dextrose prolotherapy for knee osteoarthritis with or without ACL laxity. Alt Ther Hlth Med.

[27] Reeves KD, Hassanein KM (2003). Long-term effects of dextrose prolotherapy for anterior cruciate ligament laxity. Altern Ther Health Med.

[28] Cusi M, Saunders J, Hungerford B, Wisbey-Roth T, Lucas P, Wilson S (2010). The use of prolotherapy in the sacroiliac joint. Br J Sports Med.

[29] Kim WM, Lee HG, Won Jeong C, Kim CM, Yoon MH (2010). A randomized controlled trial of intra-articular prolotherapy versus steroid injection for sacroiliac joint pain. J Altern Complement Med.

[30] Gribble PA, Delahunt E, Bleakley C (2013). Selection criteria for patients with chronic ankle instability in controlled research: a position statement of the International Ankle Consortium. J Orthop Sports Phys Ther.

[31] McGovern RP, Martin RL (2016). Managing ankle ligament sprains and tears: current opinion. Open Access J Sports Med.

[32] Cho JH, Lee DH, Song HK, Bang JY, Lee KT, Park YU (2016). Value of stress ultrasound for the diagnosis of chronic ankle instability compared to manual anterior drawer test, stress radiography, magnetic resonance imaging, and arthroscopy. Knee Surg Sports Traumatol Arthrosc.

[33] Hua Y, Yang Y, Chen S, Cai Y (2012). Ultrasound examination for the diagnosis of chronic anterior talofibular ligament injury. Acta Radiol.

[34] Wolf JM, Cameron KL, Owens BD (2011). Impact of joint laxity and hypermobility on the musculoskeletal system. J Am Acad Orthop Surg.

[35] Randolph TG, Rollins JP, Walter CK (1950). Allergic reactions following the intravenous injection of corn sugar (dextrose). Arch Surg (1920).

[36] Guharoy S, Barajas M (1991). Probable anaphylactic reaction to corn-derived dextrose solution. Vet Hum Toxicol.

[37] Lim C-Y, In J (2019). Randomization in clinical studies. Korean J Anesthesiol.

[38] Schulz KF, Grimes DA (2002). Allocation concealment in randomised trials: defending against deciphering. Lancet..

[39] Ravin TH, Cantieri MS, Pasquarello GJ (2008). Principles of prolotherapy.

[40] Hupperets MD, Verhagen EA, Van Mechelen W (2009). Effect of unsupervised home based proprioceptive training on recurrences of ankle sprain: randomised controlled trial. BMJ..

[41] Janssen KW, van Mechelen W, Verhagen EA (2014). Bracing superior to neuromuscular training for the prevention of self-reported recurrent ankle sprains: a three-arm randomised controlled trial. Br J Sports Med.

[42] Hiller CE, Refshauge KM, Bundy AC, Herbert RD, Kilbreath SL (2006). The Cumberland ankle instability tool: a report of validity and reliability testing. Arch Phys Med Rehabil.

[43] Jia Y, Huang H, Gagnier JJ (2017). A systematic review of measurement properties of patient-reported outcome measures for use in patients with foot or ankle diseases. Qual Life Res.

[44] Wang W, Liao D, Kang X (2021). Development of a valid Chinese version of the Cumberland Ankle Instability Tool in Chinese-speaking patients with chronic ankle instability disorders. Sci Rep.

[45] Janssen KW, van Mechelen W, Verhagen EA (2011). Ankles back in randomized controlled trial (ABrCt): braces versus neuromuscular exercises for the secondary prevention of ankle sprains. Design of a randomised controlled trial. BMC Musculoskelet Disord.

[46] Martin RL, Irrgang JJ, Burdett RG, Conti SF, Swearingen JMV (2005). Evidence of validity for the Foot and Ankle Ability Measure (FAAM). Foot Ankle Int.

[47] Carcia CR, Martin RL, Drouin JM (2008). Validity of the foot and ankle ability measure in athletes with chronic ankle instability. J Athl Train.

[48] Gonzalez-Sanchez M, Li GZ, Ruiz Munoz M, Cuesta-Vargas AI (2017). Foot and ankle ability measure to measure functional limitations in patients with foot and ankle disorders: a Chinese cross-cultural adaptation and validation. Disabil Rehabil.

[49] Gribble PA, Hertel J, Plisky P (2012). Using the Star Excursion Balance Test to assess dynamic postural-control deficits and outcomes in lower extremity injury: a literature and systematic review. J Athl Train.

[50] Herdman M, Gudex C, Lloyd A (2011). Development and preliminary testing of the new five-level version of EQ-5D (EQ-5D-5L). Qual Life Res.

[51] Wong EL, Ramos-Goñi JM, Cheung AW, Wong AY, Rivero-Arias O (2018). Assessing the use of a feedback module to model EQ-5D-5L health states values in Hong Kong. Patient.

[52] Wright CJ, Linens SW, Cain MS (2017). Establishing the minimal clinical important difference and minimal detectable change for the Cumberland ankle instability tool. Arch Phys Med Rehabil.

[53] Akaike H (1981). Likelihood of a model and information criteria. J Econom.

[54] Van Buuren S (2007). Multiple imputation of discrete and continuous data by fully conditional specification. Stat Methods Med Res.

[55] Rubin DB (2004). Multiple imputation for nonresponse in surveys.

[56] Whitehead J (2004). Stopping clinical trials by design. Nat Rev Drug Discov.

[57] Al-Mohrej OA, Al-Kenani NS (2016). Chronic ankle instability: current perspectives. Avicenna J Med.

