# Correction: A protocol for a randomized clinical trial assessing the efficacy of hypertonic dextrose injection (prolotherapy) in chronic ankle instability

**DOI:** 10.1186/s13063-023-07145-y

**Published:** 2023-02-13

**Authors:** Regina Wing Shan Sit, Ricky Wing Keung Wu, Samuel Ka Kin Ling, Bo Wang, Dicken Cheong Chun Chan, Benjamin Hon Kei Yip, Samuel Yeung Shan Wong, Kenneth Dean Reeves, David Rabago

**Affiliations:** 1 The Jockey Club School of Public Health and Primary Care, Chinese University of Hong Kong, Hong Kong, China; 2 The Hong Kong Insititute of Musculoskeletal Medicine, Hong Kong, China; 3 The Department of Orthopedic and Traumatology, Chinese University of Hong Kong, Hong Kong, China; 4 Roeland Park, USA; 5 Department of Family and Community Medicine, Pennsylvania State University, Hershey, USA

**Correction: Trials 23, 1063 (2022)**

**https://doi.org/10.1186/s13063-022-07037-7**

Following publication of the original article [1], the authors removed author Patrick Shu Hang Yung from the author list as he has been added by mistake.

The authorship has been updated above and the original article has been corrected.
